# No substantial excess all-cause mortality among cardiac implantable electronic device patients during the first COVID‑19 lockdown in the Leiden area

**DOI:** 10.1007/s12471-021-01650-y

**Published:** 2022-01-03

**Authors:** M. Feijen, A. D. Egorova, E. T. van der Velde, M. J. Schalij, S. L. M. A. Beeres

**Affiliations:** Department of Cardiology, Leiden Heart-Lung Center, Leiden University Medical Center, Leiden, The Netherlands

**Keywords:** Coronavirus disease 2019, Severe acute respiratory syndrome coronavirus type, Pandemic, Mortality, Cardiac implantable electronic device, Preventive measures

## Abstract

In the Netherlands, the coronavirus disease 2019 (COVID‑19) pandemic has resulted in excess mortality nationwide. Chronic heart disease patients are at risk for a complicated COVID‑19 course. The current study investigates all-cause mortality among cardiac implantable electronic device (CIED) patients during the first peak of the pandemic and compares the data to the statistics for the corresponding period in the two previous years. Data of adult CIED patients undergoing follow-up at the Leiden University Medical Centre were analysed. All-cause mortality between 1 March and 31 May 2020 was evaluated and compared to the data for the same period in 2019 and 2018. At the beginning of the first peak of the pandemic, 3,171 CIED patients (median age 70 years; 68% male; 41% ischaemic aetiology) were alive. Baseline characteristics of the 2019 (*n* = 3,216) and 2018 (*n* = 3,169) cohorts were comparable. All-cause mortality during the peak of the pandemic was 1.4% compared to 1.6% and 1.4% in the same period in 2019 and 2018, respectively (*p* = 0.84). During the first peak of the COVID‑19 pandemic, there was no substantial excess mortality among CIED patients in the Leiden area, despite the fact that this is group at high risk for a complicated course of a COVID‑19 infection. Strict adherence to the preventive measures may have prevented substantial excess mortality in these vulnerable patients.

**Supplementary Information:**

The online version of this article (10.1007/s12471-021-01650-y) contains supplementary material, which is available to authorized users.

## Introduction

The first case of coronavirus disease 2019 (COVID‑19) in the Netherlands was documented on 27 February 2020. The National Institute for Public Health and the Environment (RIVM) rapidly developed guidelines to minimise the spread and to ‘flatten the curve’. Recommendations included social distancing, general hygiene measures and home isolation [1]. Due to increasing numbers of coronavirus contaminations, an ‘intelligent lockdown’ was instituted, during which schools and restaurants were closed and the public was strongly advised to work from home. Meanwhile, the healthcare system was overloaded with COVID‑19 patients. Accordingly, non-urgent care was scaled down and outpatient medical visits were postponed, cancelled or converted to remote consultations.

The RIVM defined a number of groups at high risk for a complicated course of a COVID‑19 infection [2]. Among others, patients with chronic heart disease who qualify for an influenza vaccination were judged to be ‘high-risk patients’ and therefore strongly advised to strictly adhere to the recommendations [2]. Patients with a cardiac implantable electronic device (CIED) have chronic heart conditions and are therefore typically designated as ‘high risk’ according to the RIVM criteria [2]. Despite the ‘intelligent lockdown’, 32% excess mortality was reported in March to May 2020 across the entire Dutch population [3].

While the effects of the COVID‑19 pandemic on acute coronary syndrome patients have been extensively reported, data on the effects on chronic heart disease patients are limited [4–8]. Remote monitoring of CIED was already utilised to some extent before the pandemic. Previous studies reported a benefit of remote monitoring on healthcare utilisation and monetary costs, while data on the effect on mortality remain mixed [9, 10].

The primary objective of the current study was to investigate all-cause mortality among CIED patients during the first peak of the pandemic and to compare the data to the statistics for the same period in the two previous years. In addition, the number and type of consultations during the first peak of the pandemic were evaluated and compared to those in previous years.

## Methods

All adult CIED patients undergoing follow-up at the Leiden University Medical Centre were included in this retrospective analysis. The 2020 exposure group comprised all adult CIED patients alive on 1 March 2020 (the beginning of the first peak of the COVID‑19 pandemic in the Netherlands). The indication for a CIED was in accordance with the European Society of Cardiology (ESC) guidelines with the most prevalent indications for an implantable cardioverter-defibrillator (ICD) being primary and secondary prevention in the context of ischaemic heart disease and non-ischaemic cardiomyopathy and the most prevalent for a pacemaker being high-degree atrioventricular (AV) block and symptomatic bradycardia [11, 12]. The 2019 and 2018 control group comprised all adult patients alive on 1 March 2019 and 1 March 2018, respectively. All-cause mortality during the first peak of the COVID‑19 pandemic (1 March to 31 May 2020) was evaluated in the 2020 exposure group and compared to all-cause mortality in the control groups in the same period in 2019 and 2018.

### Data collection

Clinical data and mortality statistics were collected from the hospital’s patient information systems (EPD-Vision, Leiden, The Netherlands and Hix, Chipsoft, Amsterdam, The Netherlands). Demographic characteristics included gender, age, type of CIED, aetiology of heart disease, co-morbidities (hypertension, diabetes mellitus and prior cerebrovascular accident) and body mass index. Data on the number of outpatient clinic visits, digital or telephone contacts and CIED check-ups (physical and remote) were extracted from the patient information system.

### Endpoints

The primary endpoint was all-cause mortality in the 2020 exposure group during the first Dutch peak of the COVID‑19 pandemic (1 March to 31 May 2020), compared to the corresponding period in the 2019 and 2018 group. In addition, data on physical and remote clinical and CIED contacts were compared between the exposure and the historical control groups.

### Statistical analysis and power calculation

Minimal sample size was calculated based on mortality data among our CIED population in 2019 and 2018 and Statistics Netherlands (Centraal bureau voor de statistiek, CBS) data on excess mortality during the first peak of the COVID‑19 pandemic. CBS predicts mortality rates based on the number of deaths in the previous weeks, adjusted for seasonal effects that may have impacted mortality (e.g. influenza and weekly temperature). Excess mortality is the difference between the observed and the predicted mortality. Excess mortality in the entire Dutch population during the study period was 32%, with large variations within the country (Table S1, Electronic Supplementary Material). The provinces of Limburg and Noord-Brabant were most severely affected with excess mortality rates of 62% and 55%, respectively, while in Groningen excess mortality was only 1%. In the province of Zuid-Holland (where Leiden is situated) excess mortality during the study period was 28%. Since CIED patients are at a higher risk for a complicated course of the infection than the general population, we evaluated the hypothesis that mortality among CIED patients was at least twice as high (56%) as expected during this period. In order to obtain a power of 80% with an α of 5%, a minimum of 1,893 patients were required in the exposure group. Continuous variables are expressed as mean ± standard deviation when normally distributed, or otherwise as median and interquartile range (IQR) and were compared using the Mann-Whitney U test (non-parametric). Dichotomous variables are expressed as numbers and percentages and were compared using the chi-squared test. Statistical analysis was performed with IBM SPSS statistics (version 25) and a *p*-value < 0.05 was considered statistically significant. The institutional medical ethical committee approved the study protocol (G20.190) and waived the need for individual informed consent. All data were coded and anonymised.

## Results

### Study population

Tab. 1 shows the characteristics of the 3,171 CIED patients in the 2020 exposure group (alive on 1 March 2020). Median age was 70 years (IQR 59–78) years and 68% were male. The majority of the patients had an ICD (*n* = 2,024, 64%) and co-morbidities were highly prevalent. Most patients had an ischaemic aetiology of their heart disease (*n* = 1,296, 41%). Furthermore, 549 patients (17%) had a non-ischaemic cardiomyopathy, 179 patients (6%) a congenital heart defect and 1,147 patients (36%) a conduction disorder necessitating pacing. The characteristics of the control group (3,169 CIED patients alive on 1 March 2018 and 3,216 on 1 March 2019) were similar to those of the exposure group.

**Table 1:** Patient characteristics

		Control group		Exposure group	*p*-value
		2018	2019	2020	
		*n* = 3,169	*n* = 3,216	*n* = 3,171	
Gender (male)		2,189 (69%)	2,200 (68%)	2,154 (68%)	0.61
Age (years, IQR)		69 (59–77)	70 (59–78)	70 (59–78)	0.80
CIED type					
	Pacemaker	1,085 (34%)	1,139 (35%)	1,147 (36%)	0.27
	Implantable cardioverter defibrillator	2,084 (66%)	2,077 (65%)	2,024 (64%)	0.27
Type of heart disease					
	Ischaemic	1,326 (42%)	1,350 (42%)	1,296 (41%)	0.62
	Non-ischaemic cardiomyopathy	582 (18%)	550 (17%)	549 (17%)	0.62
	Congenital	176 (6%)	177 (6%)	179 (6%)	0.97
	Conduction disorder	1,085 (34%)	1,139 (35%)	1,147 (36%)	0.27
Co-morbidities					
	Hypertension	1,154 (46%)	1,157 (46%)	1,110 (46%)	0.96
	Diabetes	374 (14%)	365 (14%)	344 (16%)	0.32
	Cerebrovascular accident	67 (12%)	68 (12%)	76 (13%)	0.62
Risk factors					
	Active smoker	360 (15%)	365 (15%)	363 (16%)	0.42
	Body mass index (kg/m^2^, IQR)	26.2 (24–29)	26.3 (24–29)	26.3 (24–29)	0.97

*CIED* cardiac implantable electronic device, *IQR* interquartile range

### All-cause mortality

**Fig. 1:**
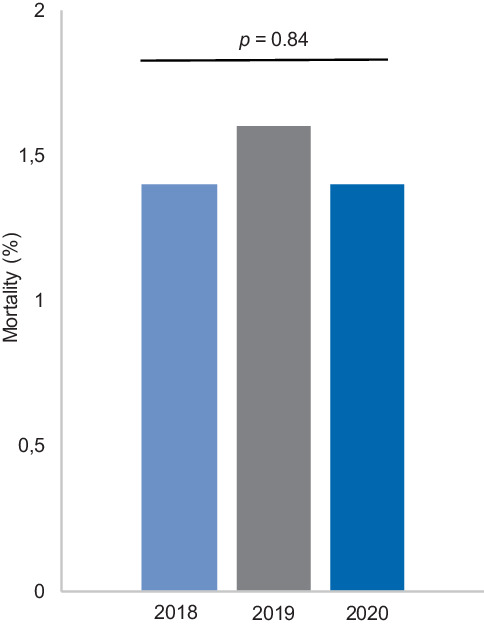
All-cause mortality (%) among cardiac implantable electronic device patients in the period 1 March–31 May 2018 (1.4%), 2019 (1.6%) and 2020 (1.4%)

During the study period, 44 of the 3,171 CIED patients in the 2020 exposure group died. Accordingly, all-cause mortality was 1.4%. All-cause mortality rates in the same period in 2019 (*n* = 50, 1.6%) and 2018 (*n* = 45, 1.4%) were comparable (*p* = 0.84, Fig. 1).

### Number of clinical contacts

**Fig. 2:**
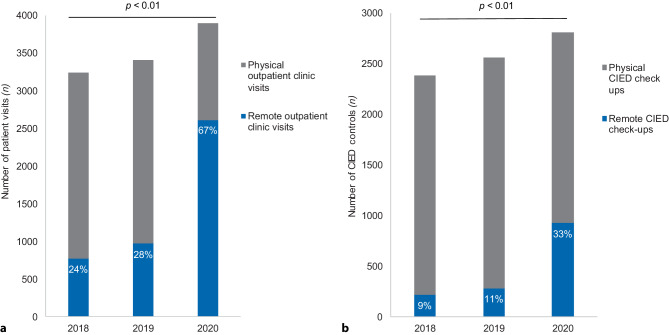
**a** Number of medical visits (cardiologist, nurse, physician assistant) from 1 March to 31 May 2018 [*n* = 3,239, 2,459 physical outpatient clinic visits and 780 (24%) remote outpatient clinic visits], and for the same period in 2019 [*n* = 3,413, 2,441 physical outpatient clinic visits and 972 (28%) remote outpatient clinic visits] and 2020 [*n* = 3,896, 1,285 physical outpatient clinic visits and 2,611 (68%) remote outpatient clinic visits]. **b** Number of cardiac implantable electronic device (*CIED*) check-ups from 1 March to 31 May 2018 [*n* = 2,383, 2,162 physical check-ups and 221 (9%) remote check-ups], and for the same period in 2019 [*n* = 2,558, 2,272 physical check-ups and 286 (11%) remote check-ups] and 2020 [*n* = 2,811, 1,882 physical check-ups and 929 (33%) remote check-ups]

During the study period in 2020, 2,611 (67%) of the outpatient clinic visits were remote (either telephone or digital assisted) and 1,285 (33%) were physical visits. There were 780 (24%) and 972 (28%) remote visits in the corresponding periods in 2018 and 2019, respectively (Fig. 2a). In 2020, 929 (33%) of CIED check-ups were remote, compared to 221 (9%) and 286 (11%) in the corresponding periods in 2018 and 2019, respectively (Fig. 2b). A significant increase in the proportion of clinical visits as well as CIED check-ups that were remote was observed between 2018 and 2020 (*p* < 0.01). A concomitant decrease in the numbers of physical visits to the hospital and in-office CIED check-ups was seen accordingly.

## Discussion

The main finding of the current study is that during the first peak of the COVID‑19 pandemic in the Netherlands, there was no substantial excess mortality among CIED patients in the Leiden area. This is despite the fact that they were at high risk for a complicated course of the infection. Specifically, the all-cause mortality rate in 2020 was similar to the rates reported for 2019 and 2018, while the RIVM reported a regional excess mortality of 28% among the population [3]. Furthermore, we observed a significant increase in remote clinical visits and CIED check-ups and a concomitant decline in physical visits and check-ups. Therefore, continuous provision of healthcare service was guaranteed.

Most recent research on cardiovascular diseases during the COVID‑19 pandemic has focussed on admission rates for acute cardiovascular problems. A striking drop in ST-elevation myocardial infarction admissions during the pandemic period has been observed in Europe and the USA [4–8]. At the same time, there was an increased incidence of out-of-hospital cardiac arrests [13, 14]. Furthermore, acute heart failure hospitalisation rates significantly declined in London, although the hospitalised patients had more severe symptoms at admission [15]. So far, little is known about how high-risk patients with a chronic cardiac disease fare during the COVID‑19 pandemic. Intuitively, an increased overall mortality would be expected.

To test this hypothesis, the current study evaluated all-cause mortality in CIED patients. The investigated patient cohort comprises relatively old patients with chronic heart disease and a CIED. The indication for a CIED was in accordance with the ESC guidelines with the most prevalent indications for an ICD and pacemaker being primary and secondary prevention in the context of ischaemic heart disease and non-ischaemic cardiomyopathy and a high-degree AV block or symptomatic bradycardia (sick sinus syndrome), respectively [11, 12]. The Dutch College of General Practitioners justifies an influenza vaccination in all patients with these underlying conditions and/or who are 60 years of age and above, thereby deeming them to be at high risk for a complicated cause of a COVID‑19 infection [16]. Furthermore, co-morbidities were highly prevalent in the study cohort. In this cohort, the present results show unaltered short-term all-cause mortality rates. Several mechanisms could have contributed to this finding. Probably, strict adherence to the preventive measures may have prevented patients from acquiring a COVID‑19 infection as well as other (seasonal) infectious diseases. In addition, based on the findings from previous studies, it could be hypothesised that reduced exposure to air pollution may have contributed to a lower excess mortality [4, 17]. During the global lockdown, for the first time since the Industrial Revolution, air pollution levels significantly decreased. The particulate matter concentration decreased by 17% across Europe and by 30% in China [18]. Fine particulate matter is transported into the systemic circulation and triggers an acute inflammatory response with increased thrombogenicity, acceleration of atherosclerosis, plaque vulnerability and increased vasoconstriction [19–22]. Short-term increased exposure (for more than 24 h) to particulate matter was associated with an increased incidence of ischaemic stroke, ischaemic heart disease, thrombosis and an increased risk of cardiovascular mortality [21, 23, 24]. In addition, it may be speculated that less vigorous physical exertion, less physiological stress and fewer accidents may have contributed to relatively lower mortality rates. Physical exercise leads to an increased propensity for thrombocyte aggregation, increased blood viscosity, enhanced thrombogenic tendency combined with elevated blood pressure and heart rate, leading to plaque erosion and subsequent acute coronary syndromes [25].

Our data also show a significant increase in the numbers of remote (either telephone or digitally assisted) outpatient clinic and remote CIED check-ups during the pandemic, with a concomitant decrease in the numbers of physical and in-office appointments despite a stable cohort size in 2018–2020. Accordingly, continuity of care was guaranteed during the pandemic and there was no deferred or scaled-down care for CIED patients. The rapid scaling up of remote monitoring was likely accompanied by several advantages. Patients were safeguarded from unnecessary exposure to COVID‑19 by not visiting the hospital [26]. Remote monitoring allows early recognition of technical issues (such as lead failure, battery depletion, programming issues including insufficient margins for sensing or capture) as well as early detection of arrhythmias [26, 27]. Furthermore, several CIED types may provide comprehensive information on worsening heart failure and thereby conceptually prevent heart failure hospitalisations [28, 29]. The current results support the previously described effectiveness of remote monitoring in lowering hospitalisation and mortality rates [30]. It is evident, however, that additional research is warranted to further unravel the underlying mechanisms, the understanding of which can help us to effectively tackle future (seasonal) epidemics and pandemics.

There are potential limitations to the present study that should be considered when interpreting the results. First, due to the retrospective study design and the current patient privacy regulations, it was not possible to further explore the cause of death. The data reflect the mortality of a large single-centre patient population. It would be of interest to confirm these observations on a nationwide level and focus on the potential regional differences. Furthermore, more insight is needed into the mechanisms leading to a relatively low short-term mortality, and the long-term effects of the pandemic remain to be investigated.

## Conclusion

During the first Dutch peak of the COVID‑19 pandemic, there was no substantial excess all-cause mortality among CIED patients in the Leiden area, despite the fact that they were at high risk for a complicated course of the infection. Strict adherence to the preventive measures and an increase in digital and remote follow-up may have potentially prevented excess mortality in this vulnerable patient group.

## Supplementary Information

Table. Regional mortality distribution from week 11 to 19* 2020
