# Supplementary material for: No substantial excess all-cause mortality among cardiac implantable electronic device patients during the first COVID‑19 lockdown in the Leiden area
Source: Neth Heart J. 2022 Jan 3;30(2):76–83. doi: 10.1007/s12471-021-01650-y (PMC8721632; doi:10.1007/s12471-021-01650-y)
Supplement: Supplementary file 1 — Table. Regional mortality distribution from week 11 to 19* 2020 [file 12471_2021_1650_MOESM1_ESM.docx]

| **Provinces** | **Excess mortality (%)** |
| --- | --- |
| Groningen | 1 |
| Friesland | 13 |
| Drenthe | 8 |
| Overijssel | 28 |
| Flevoland | 16 |
| Gelderland | 30 |
| Utrecht | 27 |
| Noord-Holland | 30 |
| Zuid-Holland | 28 |
| Zeeland | 8 |
| Noord-Brabant | 55 |
| Limburg | 62 |
| * provisional figures | |

**Supplementary material**

**Table**. Regional mortality distribution from week 11 to 19* 2020

Note. The table for regional mortality distribution from week 11 to 19 is from ‘Mortality in times of corona’, by Statistics Netherlands, 2020, <https://www.cbs.nl/en-gb/news/2020/22/mortality-in-times-of-corona> [3]
